# Leptospirosis‐induced purpura: An atypical manifestation of Weil's disease

**DOI:** 10.1002/ccr3.2692

**Published:** 2020-02-05

**Authors:** Julien Higny, Frédéric Forêt, Pierre‐François Laterre

**Affiliations:** ^1^ Intensive Care Medicine CHU UCL Namur Dinant Belgium; ^2^ Intensive Care Medicine Cliniques Universitaires Saint‐Luc Brussels Belgium

**Keywords:** coagulopathy, hepato‐nephritis, leptospirosis, purpura, Weil's syndrome

## Abstract

Purpura is a rare but documented presenting feature of severe leptospirosis. We describe a case of Weil's disease characterized by predominating coagulopathy and hepato‐nephritis. We illustrate dynamic changes in cutaneous lesions.

## Graphical abstract

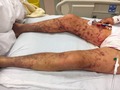

## 1 CASE PRESENTATION

**Figure 1:**
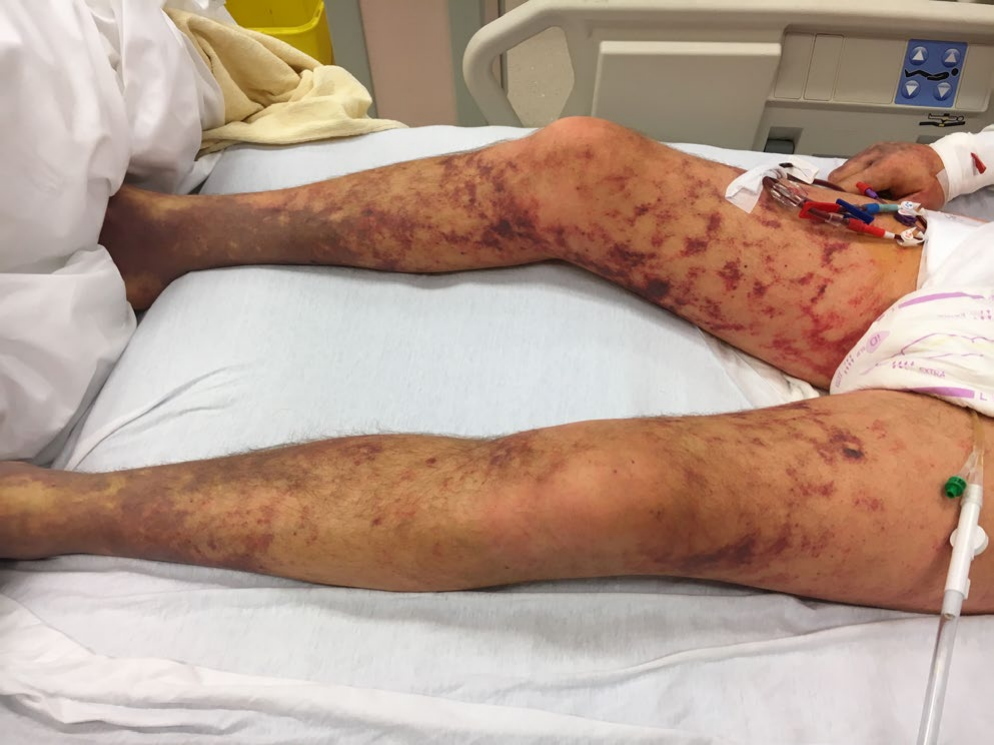
Petechiae and ecchymoses in the lower limbs of the patient (day 0)

A 49‐year‐old Dutchman who spent his winter holidays in the Belgian Ardennes was admitted to our emergency department with petechiae, ecchymoses, consciousness disorder, conjunctival suffusion, and jaundice (Figure 1). In the previous days, he had developed an acute febrile illness with myalgia, abdominal pain, diarrhea, oliguria, and headache. Invasive meningococcal infection was initially considered. Lifesaving support included blood culture, ceftriaxone, corticosteroids, fluid resuscitation, and mechanical ventilation. Blood analysis revealed an inflammatory response (CRP: 501 mg/dL) with renal failure (creatinine: 6.91 mg/dL), elevated direct bilirubin (10.19 mg/dL), leukocytosis (60 090/µL), thrombocytopenia (16 000/µL), rhabdomyolysis (CPK: 6487 UI/L), impaired coagulation (PT activity: 25%, aPTT: 67 seconds, fibrinogen: 0.94 g/L), and lactic acidosis (pH: 7.17, lactate: 10.0 mmol/L).

**Figure 2:**
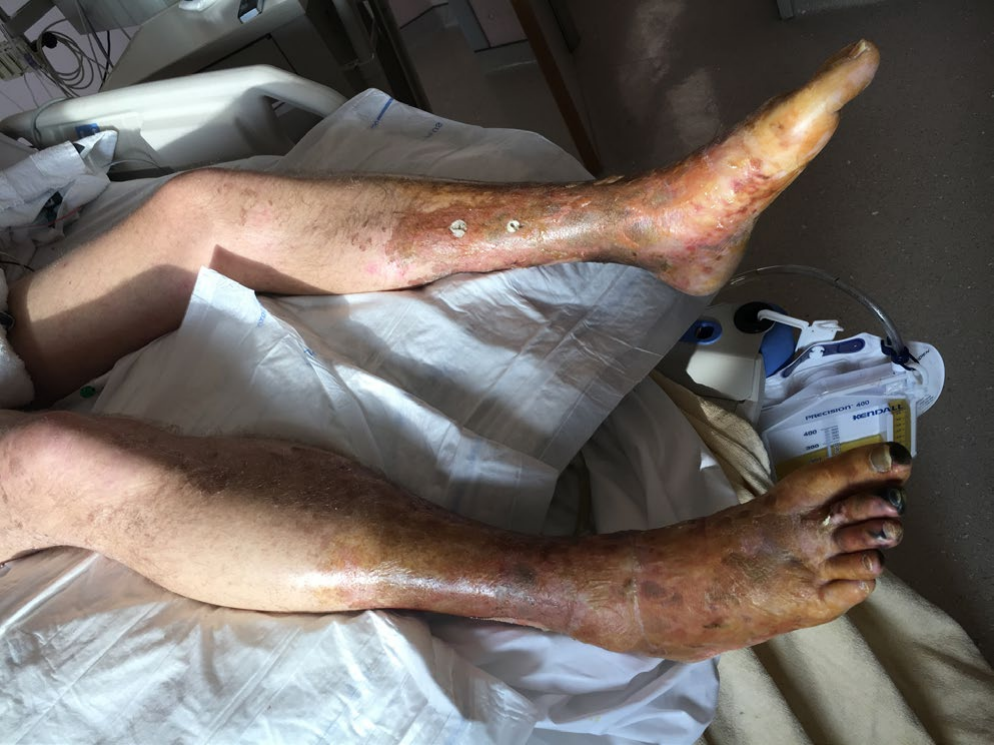
Ecchymotic purpura on the anterior surface of the legs with distal extremity necrosis (day 7)

**Figure 3:**
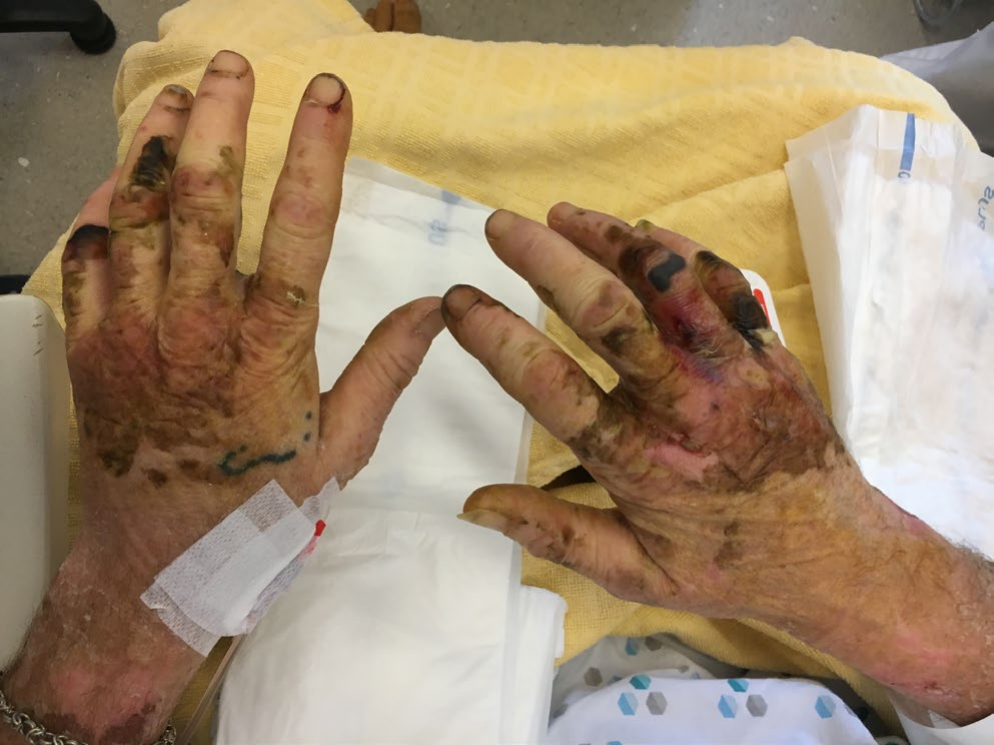
Ecchymoses and superficial necrosis on the dorsal side of the hands (day 10)

**Figure 4:**
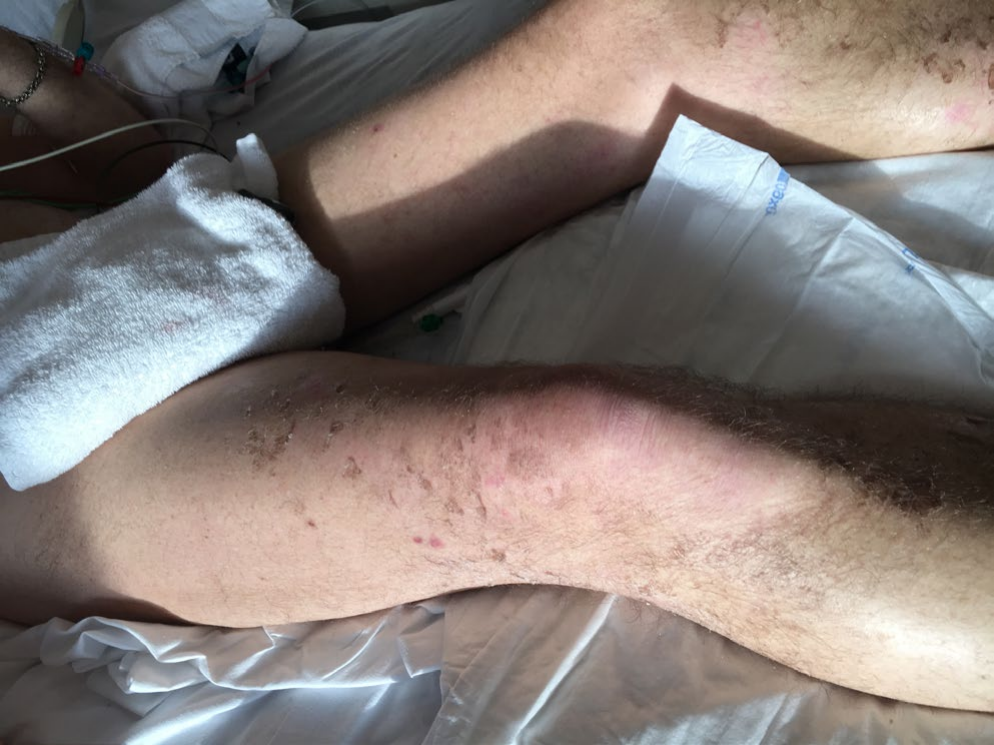
Residual cutaneous desquamations in the lower limbs (day 15)

The patient underwent continuous veno‐venous hemofiltration in the intensive care unit because of the renal failure with oliguria and lactic acidosis. Changes in cutaneous lesions are illustrated as follows: ecchymotic purpura on the anterior surface of the legs with distal extremity necrosis (Figure 2), ecchymoses, and superficial necrosis on the dorsal side of the hands (Figure 3), and residual cutaneous desquamations in the lower limbs on the 15th day (Figure 4). Because of the history of hiking around flood waters, we performed the screening test for leptospiral antibodies and started doxycycline. The complement‐fixation test with Leptospira biflexa Patoc antigen was positive.

## 2 DISCUSSION

Leptospirosis is a zoonotic disease transmitted by a spirochete. Disease in humans occurs through direct contact with infected animals or after exposure to water or soils contaminated by the urine of infected animals. Weil's disease refers to the icteric phase which may be complicated by life‐threatening conditions including renal failure, hemorrhagic diathesis, and acute respiratory distress syndrome.^1^, ^2^

## 3 CONCLUSION

We report an atypical presenting feature of severe leptospirosis which occurred during winter in a non‐tropical area. The disease was characterized by a multisystem disorder with predominating coagulopathy and hepato‐nephritis. It is uncommon for leptospirosis to present as a primary cutaneous disease. We illustrated dynamic changes in cutaneous lesions. This case is a reminder that purpura may figure as a presenting characteristic of serious leptospirosis.

## CONFLICT OF INTEREST

None declared.

## AUTHOR CONTRIBUTION

Both the authors have made substantial contribution to the preparation of this manuscript. AK: acquired the images and drafted the manuscript; DB: performed literature search and revised the manuscript for critically important intellectual content.
